# Tissue Stiffness is Not Related to Pain Experience: An Individually Controlled Study in Patients with Chronic Neck and Back Pain

**DOI:** 10.1155/2019/1907168

**Published:** 2019-12-21

**Authors:** Ann-Kathrin Lederer, Christian Maly, Tomas Weinert, Roman Huber

**Affiliations:** University Medical Center of Freiburg, Institute for Infection Prevention and Hospital Epidemiology, Center for Complementary Medicine, Freiburg, Germany

## Abstract

**Background:**

Massage therapies such as cupping are often applied in patients with chronic neck and back pain with the assumption that they can reduce increased tissue stiffness and, therefore, improve pain. The aim of this study was to clarify whether tissue stiffness is related to pain experience in patients with chronic (>3 months) back and neck pain and whether it can be altered by a cupping massage.

**Methods:**

The tissue stiffness of the point of subjectively felt maximum pain intensity of 40 patients with neck (*n* = 20) or lower back pain (*n* = 20) was measured by a myometer. Exact contralateral side served as an individual control. Side of higher stiffness was then treated with a cupping massage. 5, 10, 15, and 20 minutes as well as 24 hours after treatment, tissue stiffness was measured again. Patients rated their pain on a standardized pain questionnaire (neck pain disability score (NDI) or Oswestry disability index (ODI), respectively) before and 24 hours after treatment.

**Results:**

Compared to the contralateral control side, the more painful side did not exhibit an increased stiffness of myofascial tissue before treatment (*p*=0.827). The tissue stiffness and the side difference between treated and nontreated control sides decreased significantly after cupping (*p*=0.002 and *p*=0.001, respectively) but returned to baseline after 24 hours. NDI and ODI scores significantly decreased 24 hours after cupping (NDI: *p*=0.012, ODI: *p*=0.002).

**Conclusion:**

Tissue stiffness might not be related to pain experience in patients with chronic neck and lower back pain. Trial registration: German Clinical Trial Register (DRKS00011281).

## 1. Background

Chronic neck and back pain are common and relevant health problems [1] by affecting patients' quality of life and burdening enormous costs to the health care system [2, 3]. The origin of chronic nonspecific back pain is typically assumed to be multifactorial, with a strong biopsychosocial component [4, 5]. Patients and physicians often consider “muscle stiffness” a main factor for neck and back pain [6, 7]. The definition of “muscle stiffness” is inconsistent as some refer to stiffness as limited range of motion, but others define stiffness as muscular hypertonicity or as hardening of myofascial tissue. The stiffness of the myofascial tissue is a functional parameter, which can be approximated with a myometer, a small hand-held device [8, 9]. It is reported that a myometer might be able to assess mechanical properties similar to others methods such as sonoelastography in patients with neck and back pain [10–12]. According to the assumption that changing of tissue stiffness leads to pain, tissue manipulation is a frequently used manual therapy approach for treatment of musculoskeletal pain [13] and has, in clinical studies, been efficacious to improve pain [14]. One kind of physical manipulation is cupping, which is a well-defined technique for physical therapy of the back [15]. Cupping has traditionally been used in European, Asian, and Arabic medicine and is nowadays mostly used for treatment of chronic pain of the musculoskeletal system [15]. Several controlled studies showed efficacy of cupping in patients with back pain [16–19]. During cupping massage, the skin and subjacent tissue is sucked by vacuum into a cupping glass, which leads to an irritation of the tissue [20, 21]. Due to stop cock equipped cupping glasses, cupping offers the advantage of controlling treatment pressure in comparison to other physical therapies. When the pressure in the cupping glass is measured, which has been done under experimental conditions by our group before, cupping can be standardized very well [21]. Whether pain improvement after physical manipulation such as cupping is related to decrease of tissue stiffness has, however, not been investigated. Therefore, the aim of our individually controlled study was to clarify whether myofascial stiffness, measured by a myometer, is related to pain experience in patients with chronic back and neck pain and whether it can be altered by a cupping massage.

## 2. Methods

### 2.1. Design

The monocentric, individually controlled, experimental trial was performed between November 2016 and January 2017, reported according to the CONSORT guidelines [22]. It was registered at the German Clinical Trial register (DRKS00011281) and approved by the ethical committee of the University Medical Center of Freiburg, Germany (EK Freiburg 387/16) before onset. The study was performed according to the principles of the declaration of Helsinki and to the guidelines of ICH for a good clinical practice (GCP). All patients gave their written informed consent before participation. All data were handled strictly confidential.

Measurement of all patients was performed by only one skilled investigator (CM) under supervision of another skilled investigator (RH) at University Medical Center Freiburg.

### 2.2. Participants

Patients were recruited via newspaper announcement. Interested patients were screened for eligibility by phone call and, if eligible, invited for a personal examination the following days. All patients were intensively informed about the aim and the implementation of the study before signing written informed consent. Eligible for the study were patients between 18 and 60 years of age with chronic (>3 months) nonspecific pain of the neck or lower back [5] and a minimum score of 15 points (out of 50 points) in the neck pain disability score (NDI) [23] or Oswestry disability index (ODI) [24, 25] at the day of screening and inclusion, respectively. Detailed medical history of all patients was obtained, and neurological and physical examinations were performed by a trained physician before inclusion. Any sign of specific back pain (abnormalities in muscle reflexes, sensitivity, muscular strength, progressive pain, tenderness upon percussion) were exclusion criteria. Further criteria of exclusion were comorbidities like advanced heart failure, liver cirrhosis, psychiatric disorders or cancer, skin diseases on the back, urticaria by heat, blood coagulation disorders or intake of anticoagulants, pregnancy or lactation, inability to speak or understand German, participation in another clinical trial in the last 4 weeks, and self-reported drug abuse. No other physical treatment was allowed during the study period. Patients had to avoid muscle relaxants and pain medication during the trial.

### 2.3. Measurement of Tissue Stiffness

**Figure 1:**
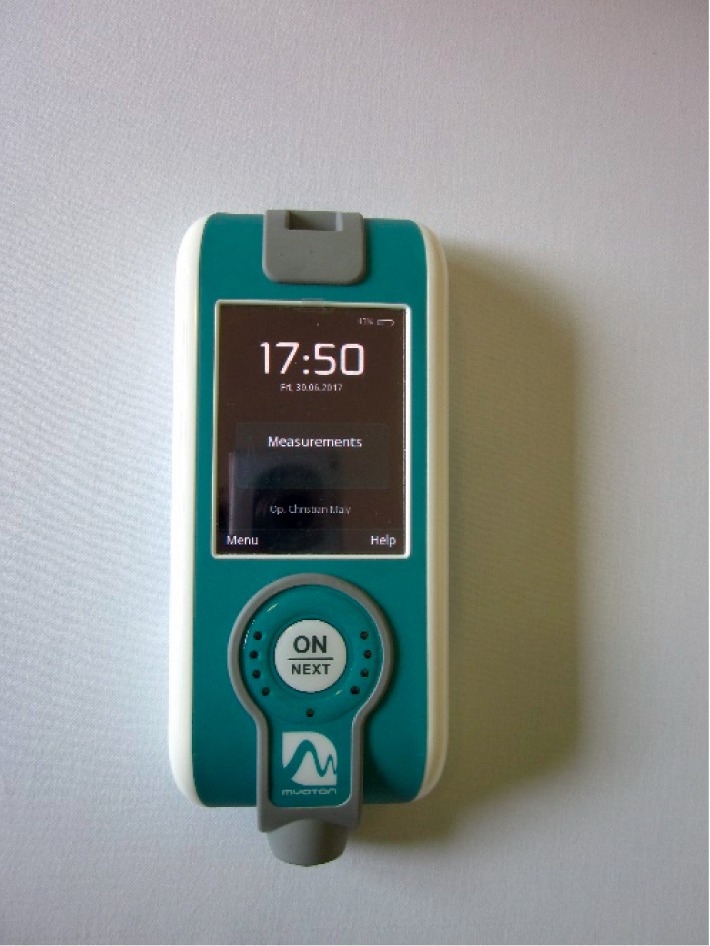
Tissue stiffness as well as oscillation frequency and logarithmic decrement were measured with the myometer MyotonPro® (MyotonAS, Tallinn, Estonia).

Tissue stiffness was measured with the myometer MyotonPro® (MyotonAS, Tallinn, Estonia) (Figure 1). The method is noninvasive and fast and has been shown to be highly reproducible independent of the investigator [9, 26–28]. The method has been validated before onset by self-experiments [29]. The reproducibility of myometrical measurement was tested by several measurements of different muscles (Quadriceps femoris and trapezoid) of the same participant without physical manipulation. Following, aiming to find the best place and time for myometrical measurement and to ensure reproducibility, further measurements of the back before and after cupping were performed. The interinvestigator reproducibility was excellent (variation <10%) when exactly the same point of the skin was used [30].

After putting the probe of the device with a size of 1 × 1 mm on the surface of the skin, it applies a mechanical impulse with a prepressure of 0.18 N. The aim of the prepressure is compression of subcutaneous tissue. It is followed by a quick mechanical impulse with a pressure of 0.40 N. Consequence is a natural oscillation of the fascia, which is recorded by the accelerometer of the device. The device automatically calculates arithmetic means of 5 single measures of dynamic stiffness, oscillation frequency, and logarithmic decrement of the tissue [8].

During the whole trial, the point of measurement was the place of subjectively felt maximum pain intensity on the neck or back before treatment, which was marked with a skin-friendly and water-proofed pen. The place of subjectively felt maximum pain intensity was chosen due to the hypothesis that it might be the place of highest stiffness. An additional mark was set on the exactly contralateral side of the neck or back. Tissue stiffness, oscillation frequency, and logarithmic decrement were measured at both sides. Measurement was performed before intervention and 5, 10, 15, and 20 minutes as well as 24 hours after intervention.

### 2.4. Cupping

**Figure 2:**
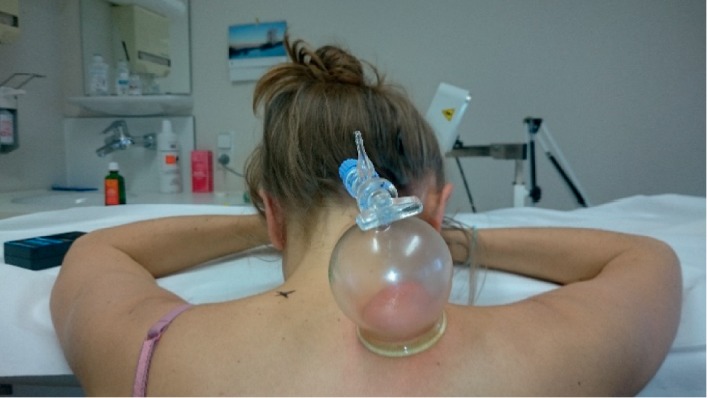
Procedure of cupping of patients with chronic neck pain: the image shows the sucking of tissue into a cupping glass, and skin is reddened after massage. The patient is seated lying head stress-free on a prepared shelf. The side of higher tissue stiffness was treated with cupping massage. The point of maximum pain and the corresponding contralateral side are marked with an “*x*” (one is not visible due to the cupping glass).

Due to the hypotheses that higher tissue stiffness correlates with more pain and cupping would reduce tissue stiffness, the side with the higher stiffness was chosen for intervention. All patients were seated putting their head stress-free on a prepared shelf (Figure 2). After applying massage oil to the skin, a cupping glass was prepared with a stop cock, an opening diameter of 5 cm, and a volume of 168 ml (Figure 2) was evacuated by holding a burning alcohol soaked cotton swab close to the opening for a second and immediately placed in a distance of about 3 cm near to the mark on the back or neck of the patient [30]. An area of 2 cm around the mark was not treated because cupping-induced swelling of the skin and subcutis might have interfered with the measurement of tissue stiffness [20]. The glass was moved gently up and down for five minutes to perform a one-sided back massage in a field of about 20 × 20 cm (Figure 2) [30]. In order to ensure reproducibility of our experiments, pressure was measured in all patients by manometer 30 seconds after placing the cupping glass on the skin.

### 2.5. Questionnaires

All patients had to answer the NDI or ODI as standardized and validated questionnaires for neck pain and back pain, respectively, before and 24 hours after cupping.

The NDI as well as the ODI captures 10 dimensions of neck-specific disability (pain intensity, personal care, lifting, reading, headache, concentration, work, driving, sleeping, and recreation). Each dimension is assessed with 1 item, measured on a scale ranging from “no disability” (=0 points) to “full disability” (=5 points) [23, 25]. A maximum of 50 points can be achieved.

### 2.6. Outcome Measures

Primary outcome was the proportion of concordance between subjectively felt maximum pain and higher myometrically measured tissue stiffness in this area compared to tissue stiffness of the contralateral area of less pain. Secondary targets were the course of tissue stiffness 5, 10, 15, and 20 min as well as 24 hours after cupping in comparison to the tissue stiffness of the nontreated side. NDI and ODI after 24 hours in comparison to baseline and in relation to tissue stiffness were further secondary targets.

### 2.7. Planning of Sample Size and Data Analysis

The study was planned as an explorative trial. Considering a statistical power of 80% and an expected medium effect size (*d* = 0.66), it was calculated that 40 patients (20 patients with neck pain and 20 patients with lower back pain) were needed to detect a statistical difference of *p* < 0.05 for correlation of stiffness and pain intensity within the groups.

The results were collected in a predesigned table. IBM SPSS (version 21.0) was used for analysis. *p* < 0.05 was considered significant. Results were checked for normal distribution. Pearson's correlations coefficient was used to describe the relation between tissue stiffness and pain intensity. A two-tailed *t*-test was performed to calculate differences between the points of measurement. A one-factorial variance analysis was performed to investigate the change of stiffness before and after physical manipulation.

## 3. Results

40 patients were included in the study and analyzed (Table 1); there was no drop out. Patients were on average 48 ± 9 years old, and 78% of patients were female with an average BMI of 24 ± 4 kg/m^2^. They suffered from neck or back pain for almost 9 years (mean 107 ± 141 months). 20 patients had chronic nonspecific neck pain (49 ± 8 years, ♀ 90%, BMI 26 ± 5 kg/m^2^, suffering for 129 ± 169 months) and 20 patients had chronic nonspecific pain of the lower back (47 ± 10 years, ♀ 65%, BMI 22 ± 2 kg/m^2^, suffering for 85 ± 118 months). All patients were able to indicate a side (left or right) with dominant pain.

### 3.1. Primary Research Question

Only 45% (*n* = 9) of the patients with neck pain and 50% (*n* = 10) of the patients with lower back pain located their subjectively felt maximum of pain intensity on the side with higher tissue stiffness. 55% of the neck pain patients and 50% of the patients with lower back pain had higher tissue stiffness on the control side which was less painful. There was no correlation between the side of maximum pain and tissue stiffness (*r* = 0.036, *p*=0.827).

### 3.2. Course of Tissue Stiffness after Cupping

**Figure 3:**
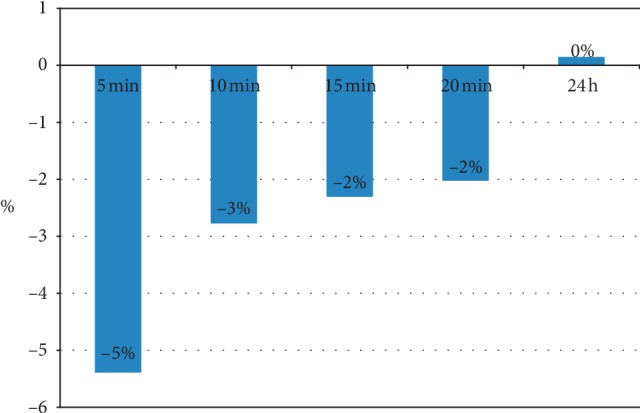
Patients with low back pain (*n* = 20) showed decrease of tissue stiffness after cupping, which was completely recurrent after 24 hours (percentage change compared to baseline stiffness).

**Figure 4:**
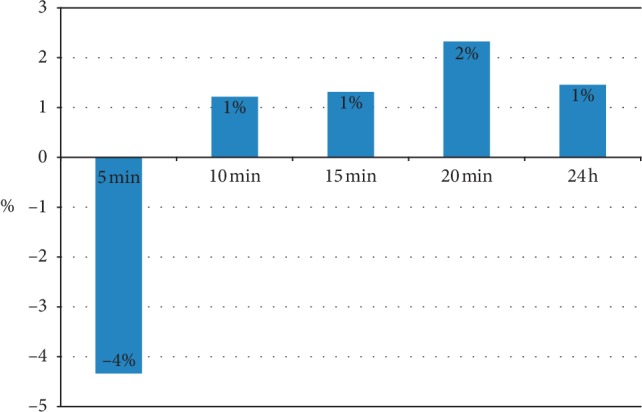
Patients with neck pain (*n* = 20) showed an initial, nonlasting decrease of tissue stiffness 5 minutes after cupping (percentage change compared to baseline stiffness before cupping).

On the treated side tissue stiffness slightly decreased after cupping compared to baseline (see Table 2), the differences were only significant 5 minutes after intervention (*p*=0.002). Tissue stiffness returned to baseline values after 24 hours (see Figures 3 and 4 as well as Table 2).

### 3.3. Questionnaires

The mean score of NDI/ODI decreased significantly from 17 (range 15–27) to 15 (range 2–26) points 24 hours after cupping (*p* < 0.001, *d* = 0.67). The mean baseline score of both subgroups is shown in Table 1. Patients with neck pain had a mean NDI score of 18 (range 15–21) points before and of 15 (range 10–21) after cupping (*p*=0.012, *d* = 0.62). Patients with lower back pain had a mean ODI score of 16 (range 14–18) before and 14 (range 12–17) after treatment (*p*=0.002, *d* = 0.79).

## 4. Discussion

This study is the first, investigating the relation between pain experience and tissue stiffness in patients with chronic back and neck pain. The results indicate that the intensity of pain might not be related to an increased stiffness of the myometrically measurable myofascial tissue. Almost 50% of the patients had the higher baseline tissue stiffness on the side with less pain. The performed physical manipulation altered tissue stiffness over the most painful region in participants with chronic neck and back pain just for a short time. 24 hours after intervention, the tissue stiffness was as before, while patients' pain ratings 24 hours after manipulation improved substantially. To date, less is known about the relation between the configuration of myofascial tissue and patients' pain experience. Park et al. compared the tissue stiffness of patients with cervicogenic headache with healthy volunteers using the same myometer as in our study. They found a higher tissue stiffness in headache patients [10], but they did not perform intraindividual comparisons as in our study and the results are, therefore, at least not contradictory to ours. Another study found a relationship between fascial length measured by MRI and high-intensity pain in 72 patients with chronic back pain [31]. The authors assume that a shorter fascial length might predispose for increased pressure of the paraspinal compartment and subsequent pain. Another study showed a decrease of the cross-sectional area of multifidus and erector spinae muscles in patients with chronic back pain [32]. However, nothing is yet known about the relation between fascial length or cross-sectional muscle area and stiffness of tissue. Hence, a comparison of the results with our results is not possible; the pathogenic importance of tissue stiffness has to be evaluated more thoroughly.

Our results suggest that pain relief after cupping massage cannot be explained by reducing tissue stiffness. Pain experience underlies a complex system which includes endogenous analgesic substances, internal neuronal networks, and emotional and social factors [33]. Counter irritation with different somatic sensory stimuli can relieve pain [34], due to modulation of the neuronal network [35, 36]. Pain improvement by cupping seems, by these hypotheses, to be induced on a more systemic level. But how these neuronal pathways interact with physical therapies in detail has still to be clarified.

### 4.1. Limitations

Strength of our study is the high level of standardization regarding the setting (monocentric, always the same investigator, follow-up to the same time of the day) and the interventions (defined by measuring the pressure and surface area). A limitation is the lack of blinding, which was not possible due to the nature of the intervention. The study focused on the myometrically measurable tissue stiffness at one single spinal level. Therefore, nothing is known about the pain of other spinal levels as well as the relation between pain and other types of stiffness, which are not measurable by a myometer. Furthermore, the most painful single point, which was chosen for measurement, might not be the point of pathophysiology as it is known that pain and dysfunction can occur far from tissue irritation. A parallel group design or a crossover trial would have been other options to analyze the effect of cupping on tissue stiffness, but we expected high variations when measuring at different days or when comparing different individuals and, therefore, decided to prefer an intraindividual comparison of two laterally reversed sides. Finally, 40 study participants, especially with the numerous exclusion criteria, do not necessarily represent the many different types and variations of conditions that can cause neck and back pain. It is still possible that some persons exhibit tissue stiffness changes that could be measured using this method and that this study failed to recruit enough (or any) to demonstrate it or that using different sites (not based on most painful region) could have yielded different results.

## 5. Conclusions

Our study gives new insights into the relation between the myometrically measurable tissue stiffness and pain. This study found no relation between the most painful site and tissue stiffness as measured with a myometer in 40 participants with chronic neck and back pain. It also found that tissue stiffness as measured with a myometer did not change following cupping massage applied at the most painful site. The results of the study should lead to further, confirmatory research.

## Figures and Tables

**Table 1:** Description of included patients.

	Neck pain (*n* = 20)	Low back pain (*n* = 20)	Total (*n* = 40)
Age (years ± SD)	49.1 ± 7.8	47.4 ± 9.9	48.3 ± 8.8
Gender (male/female %)	10/90	35/65	22.5/77.5
Body mass index (kg/m^2^ ± SD)	26.2 ± 4.6	22.2 ± 2.2	24.2 ± 4.1
Duration of symptoms (months ± SD)	129 ± 169	85 ± 118	107 ± 141
Baseline NDI (points; range)	18 (15–27)		
Baseline ODI (points; range)		16 (14–18)	
Pressure in the cupping glass (mbar ± SD)	274 ± 34	281 ± 27	278 ± 31

SD = standard deviation.

**Table 2:** Course of stiffness of cupped side, noncupped side, and difference of both.

Stiffness (N/m) ± SD	Neck pain (*n* = 20)	Low back pain (*n* = 20)	Total (*n* = 40)
Mean difference of both sides (cupped—control side)			
Baseline	56 ± 52	79 ± 59	68 ± 56
5 min	25 ± 52	43 ± 52^*∗*^	34 ± 52^*∗*^
10 min	19 ± 66	40 ± 75	39 ± 71
15 min	25 ± 55	30 ± 70	28 ± 63
20 min	31 ± 61	35 ± 77	33 ± 69
24 h	36 ± 68	47 ± 81	42 ± 75

Mean of cupped side			
Baseline	374 ± 117	510 ± 94	442 ± 211
5 min	350 ± 73	483 ± 92^*∗*^	417 ± 83^*∗*^
10 min	353 ± 79	495 ± 100	424 ± 90
15 min	369 ± 79	496 ± 91	433 ± 85
20 min	375 ± 88	498 ± 92	437 ± 90
24 h	378 ± 118	511 ± 108	445 ± 113

Mean of control side			
Baseline	341 ± 101	430 ± 109	386 ± 105
5 min	343 ± 93	439 ± 91	391 ± 92
10 min	355 ± 76	455 ± 108	405 ± 92
15 min	350 ± 85	465 ± 102	408 ± 94
20 min	348 ± 84	463 ± 104	406 ± 94
24 h	353 ± 91	463 ± 114	408 ± 103

^*∗*^Significant reduction (*p* ≤ 0.05 compared to baseline).

## Data Availability

The data used to support the findings of this study are available from the corresponding author upon request.

## Abbreviations

MRI: Magnetic Resonance Imaging
NDI: Neck pain disability score
ODI: Oswestry disability index.
